# 

**DOI:** 10.1186/1475-925X-13-S1-I1

**Published:** 2014-02-28

**Authors:** Tuan D Pham, Xiaoyi Jang, Kazuhisa Ichikawa

**Affiliations:** 1 Aizu Research Cluster for Medical Engineering and Informatics, Center for Advanced Information Science and Technology,The University of Aizu, Aizu-Wakamatsu, Fukushima 965-8580, Japan; 2 Department of Computer Science, University of Münster, Einsteinstrasse 62, 48149 Münster, Germany; 3 Division of Mathematical Oncology, The Institute of Medical Science, The University of Tokyo, 108-8639, Tokyo, Japan

## 

The theme of this special issue (supplement) is "*Current Challenging Medical Image Analysis"*. This issue consists of two selected papers presented at the 35^th ^IEEE-EMBC Workshop on *Current Challenging Image Analysis and Information Processing in Life Science*, which was held on 03 July 2013, Osaka, Japan; and one invited paper to reflect the theme of this issue. All three papers were accepted based on the peer-review process of *BioMedical Engineering Online *journal.

The first paper entitled "*Deformable part models for object detection in medical images*" by Klaus Toennies, *et al*., presents a novel application of a deformable model of the finite element method for the detection of objects in medical images. This proposed approach is promising for context-based detection, model-based segmentation, and shape analysis of medical objects.

The second paper entitled "*Motion correction of whole-body PET data with a joint PET-MRI registration functional*" by Michael Fieseler, *et al*., makes use of the multi-modal information simultaneously provided by both PET (position emission tomography) and MRI (magnetic resonance imaging) data for the registration and correction of respiratory motion, whereas current methods for PET-MRI-based motion correction do not utilize the valuable sources of information provided by PETdata.

The last paper entitled "*Automated CT detection of intestinal abnormalities and ischemia for decision making in emergency medicine*" by Taichiro Tsunoyama, *et al*., reports an original work on automated image analysis of bowel ischemia and abnormality patterns, which is important for the urgent treatment of patients in emergency departments. The combination of the sources of knowledge from medicine and imaging science offers a computer-aided tool that can be essential to surgeons to gain insights into this complex disease.

Many thanks are to Professor Kenneth R. Foster, the Editor-in-Chief of *BioMedical Engineering Online *for his approval of the proposal of the publication of this special issue. It was a great pleasure to work with Ms. Sarah Headley of BioMed Central, who kindly and frequently communicated with the Guest Editor in all editorial aspects to help ensure the editing quality and timely publication of this special issue.

December 2013

Tuan D. Pham

Guest Editor

## Competing interests

The Supplement Editors declare that they have no competing interests.

## Declarations

This article has been published as part of *BioMedical Engineering OnLine *Volume 13 Supplement 1, 2014: Selected articles from the 35th Annual International Conference of the IEEE Engineering in Medicine and Biology Society: Workshop on Current Challenging Image Analysis and Information Processing in Life Sciences. The full contents of the supplement are available online at http://www.biomedical-engineering-online.com/supplements/13/S1

